# Role of Protein Tyrosine Phosphatase Receptor Type E (PTPRE) in Chemoresistant Retinoblastoma

**DOI:** 10.3390/ijms25084572

**Published:** 2024-04-22

**Authors:** Lars Mohren, Annika Doege, Natalia Miroschnikov, Oliver Dräger, Maike Anna Busch, Nicole Dünker

**Affiliations:** 1 Institute for Anatomy II, Department of Neuroanatomy, Center for Translational Neuro- and Behavioral Sciences (C-TNBS), Medical Faculty, University of Duisburg-Essen, 45122 Essen, Germany; lars.mohren@uk-essen.de (L.M.); annika.doege@uk-essen.de (A.D.); nicole.duenker@uk-essen.de (N.D.); 2 Department of Medical Oncology, Sarcoma Center, West German Cancer Center, Medical Faculty, University of Duisburg-Essen, 45122 Essen, Germany; 3 Medical School OWL, Cellular Neurophysiology, Bielefeld University, 33615 Bielefeld, Germany; oliver.draeger@uni-bielefeld.de

**Keywords:** PTPRE, miR631, retinoblastoma, CAM assay, etoposide, chemoresistance, tumorigenesis

## Abstract

Protein tyrosine phosphatase receptor type E (PTPRE) is a member of the “classical” protein tyrosine phosphatase subfamily and regulates a variety of cellular processes in a tissue-specific manner by antagonizing the function of protein tyrosine kinases. PTPRE plays a tumorigenic role in different human cancer cells, but its role in retinoblastoma (RB), the most common malignant eye cancer in children, remains to be elucidated. Etoposide-resistant RB cell lines and RB patients display significant higher PTPRE expression levels compared to chemosensitive counterparts and the healthy human retina, respectively. PTPRE promotor methylation analyses revealed that PTPRE expression in RB is not regulated via this mechanism. Lentiviral PTPRE knockdown (KD) induced a significant decrease in growth kinetics, cell viability, and anchorage-independent growth of etoposide-resistant Y79 and WERI RB cells. Caspase-dependent apoptosis rates were significantly increased and a re-sensitization for etoposide could be observed after PTPRE depletion. In vivo chicken chorioallantoic membrane (CAM) assays revealed decreased tumor formation capacity as well as reduced tumor size and weight following PTPRE KD. Expression levels of miR631 were significantly downregulated in etoposide-resistant RB cells and patients. Transient miR631 overexpression resulted in significantly decreased PTPRE levels and concomitantly decreased proliferation and increased apoptosis levels in etoposide-resistant RB cells. These impacts mirror PTPRE KD effects, indicating a regulation of PTPRE via this miR. Additionally, PTPRE KD led to altered phosphorylation of protein kinase SGK3 and—dependent on the cell line—AKT and ERK1/2, suggesting potential PTPRE downstream signaling pathways. In summary, these results indicate an oncogenic role of PTPRE in chemoresistant retinoblastoma.

## 1. Introduction

Retinoblastoma (RB) is a malignant tumor that arises in the developing retina of children, affecting approximately 1 in 16,000 live births [1,2,3]. In more than 95% of the cases, the tumor initially develops through biallelic loss of the tumor suppressor gene *RB1* and further progresses after additional genetic/epigenetic changes (for review: [1]). RB is curable, but tumors become advanced if treatment is delayed, hindering vision and globe salvages with a risk of metastasis [4]. VEC (vincristine, etoposide, carboplatin) combination chemotherapy is commonly used for globe sparing with different routes of drug delivery [3,5]. However, RB treatment is often limited and side effects of chemotherapeutics as well as acquired drug resistances lead to relapses or secondary carcinomas [6]. Besides, similar to other cancers, therapeutics selectively targeting the tumor while sparing the normal retina are desired.

Protein tyrosine phosphatases (PTPs) working in concert with protein tyrosine kinases (PTK) to control cellular homeostasis are novel targets for cancer drug discovery. Cell signaling is tightly controlled by a balance between pathway activators like PTKs, and inactivators like PTPs [7,8]. Aberrant regulation of protein phosphorylation on tyrosine residues is a well-established cause of cancer [9,10]. Receptor-type protein tyrosine phosphatases (PTPRs) are a subgroup of PTPs comprising 21 members that share a transmembrane domain (for review: [7]). Receptor-type protein tyrosine phosphatase epsilon (PTPRE), a member of the PTPR family, has two isoforms, a membrane type PTPRE (memPTPRE) that anchors in the cell membrane and is highly expressed in brain, testes, lymph node and lung and a cytosol type PTPRE (cytPTPRE), which is mainly localized in cytosol and is found in the spleen, lung and thymus [10]. Alterations in the expression of these phosphatases, e.g., by promoter methylation, cause aberrations in the activation of crucial cellular pathways like proliferation, apoptosis, survival, adhesion, migration, and invasion, suggesting that PTPRs are critical components in malignant transformation and tumorigenesis [7,10]. In general, PTPs have been controversially discussed as either tumor suppressors or oncogenes [7,11,12]. Upregulated levels of PTPRE have been found in several cancer entities, including acute myeloid leukemia and renal cell carcinoma [13,14]. It has been shown that promoter methylation is an important mechanism to regulate PTPR expression (for review; [7]) and aberrant PTPRE promoter methylation has been observed in various cancers [15,16]. Furthermore, PTPRE has been described as a downstream target of miR631 in hepatocellular carcinoma [17] and an up-regulation and activation of PTPRE has been observed in MCF-7 cells and MDA-MB-231 upon stimulation with fibroblast growth factor (FGF) [18]. At the downstream signaling level, receptor-type PTPRE dephosphorylates and thereby activates Src in murine mammary tumor cells, which in turn contributes to oncogenesis [9,10,19,20]. Besides, PTPRE contributes to ERK1/2 and AKT activation in human breast cancer cells [18] and in mice PTPRE antagonizes the activation of voltage-gated potassium channels by tyrosine kinases like Src [21].

Studies of PTPRE signaling in cancer in general and in RB in particular will help to develop novel RB treatment strategies targeting this tyrosine phosphatase and its up- and downstream signaling components. Thus, in the study presented, we set out to unravel the expression profile of PTPRE in chemosensitive and chemoresistant RB cell lines and patient tumors and its potential regulation by promotor methylation, miR631 targeting, and FGF signaling. Besides, the effects of a lentiviral PTPRE knockdown on survival, growth and re-sensitization as well as PTPRE downstream signaling of etoposide-resistant RB cells were investigated in vitro and in vivo. Data gained by our analyses revealed decreased cell viability and tumor development as well as re-sensitization towards etoposide upon PTPRE KD in RB cells, indicating an oncogenic role of PTPRE in chemoresistant retinoblastoma, involving miR631 and SGK3 regulating mechanisms.

## 2. Results

### 2.1. PTPRE Is Differentially Expressed in Retinoblastoma Cell Lines and Patient Tumors

**Figure 1:**
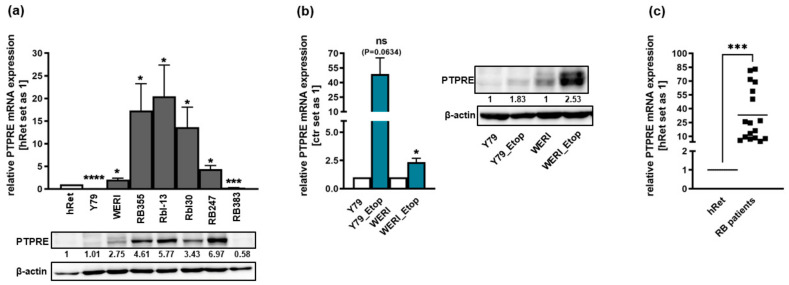
Endogenous PTPRE expression in seven chemosensitive (**a**) and the etoposide-resistant (Etop) retinoblastoma cell lines Y79_Etop and WERI_Etop (**b**) as well as RB patient tumor specimen (**c**) as revealed by Western blot (**a**,**b**) analyses and quantitative Real-time PCR (**c**). Ctr: chemosensitive Y79 and WERI RB control cells. _Etop: etoposide-resistant Y79_Etop and WERI_Etop RB cells indicated numbers that represent intensity ratios normalized to ß-actin, used as a loading control (**a**,**b**), calculated using Micro Manager 1.4 software. (**c**) PTPRE expression levels in enucleated RB patient eyes as compared to the healthy human retina (hRet). Values are means of at least three independent experiments ± SEM. ns not significant; * *p* < 0.05; *** *p* < 0.001; **** *p* < 0.0001 statistical differences compared to the control group calculated by Student’s *t*-test.

We analyzed the expression of PTPRE in the parental, chemosensitive RB suspension cell lines Y79, WERI, Rbl-13, Rbl30, RB247 and RB383 as well as in the adherent cell line RB355. Compared to the healthy human retina, PTPRE was differentially expressed with significantly higher mRNA levels in WERI, RB355, Rbl-13, Rbl30 and RB247 cells and significantly decreased expression in Y79 and RB383 cells (Figure 1a). Exemplary Western blot analysis mainly confirmed this expression pattern at the PTPRE protein level, except for Y79 (Figure 1a). In addition, compared to chemosensitive counterparts, we found an increase in PTPRE expression in the etoposide-resistant RB cell lines Y79_Etop and WERI_Etop; however, full significance was only reached in the latter cell line (Figure 1b). Western blot analysis confirmed this expression pattern at the PTPRE protein level (Figure 1b). Moreover, significantly increased PTPRE mRNA and protein expression levels were detected in RB patient tumors compared to the healthy human retina (Figure 1c).

**Figure 2:**
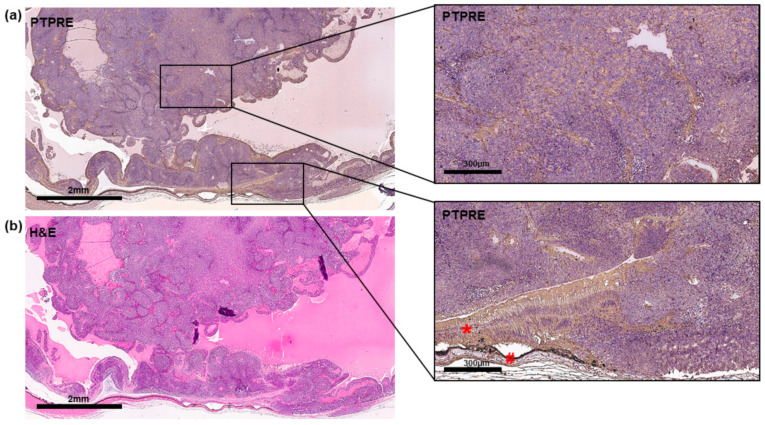
PTPRE expression in a primary RB patient tumor. (**a**) Immunohistochemical analysis of PTPRE expression (brown signal) in an exemplary paraffin section of a human RB patient tumor counterstained with hematoxylin (blue nuclei staining). (**b**) Corresponding hematoxylin and eosine (H&E) stain. Scale bars, 2 mm and 300 µm (magnified insets), *: retina and #: pigment epithelium.

The PTPRE expression pattern in an RB patient tumor was revealed by immunohistochemical staining and is shown in Figure 2.

### 2.2. PTPRE Expression Is Not Regulated by Promotor Methylation

**Figure 3:**
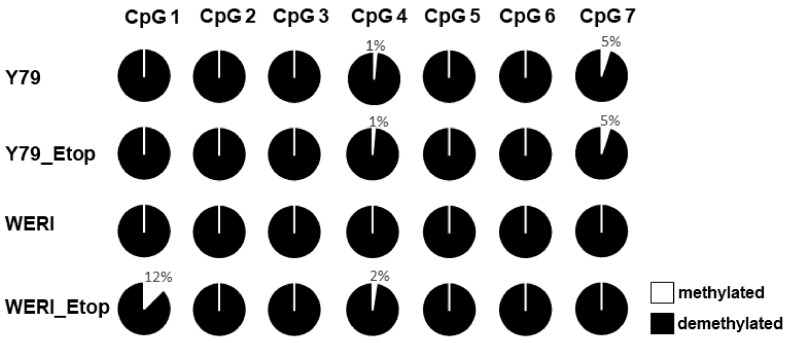
Genomic bisulfite sequencing of the PTPRE promoter associated CpG sites in etoposide-resistant (Etop) and chemosensitive retinoblastoma cell lines. Each circle represents a single CpG site. The average methylation of all CpG dinucleotides analyzed is indicated based on sequencing of at least ten clones. Nearly all CpGs in the PTPRE promoter of the etoposide-resistant (Etop) and chemosensitive RB cells analyzed were demethylated.

Since an epigenetic regulation of PTPRE via promotor methylation has been described for pituitary adenomas and papillary thyroid carcinoma [15,16], we performed PTPRE promotor methylation analyses for the etoposide-resistant RB cell lines Y79 and WERI and their chemosensitive counterparts. Bisulfite conversion and subsequent sequencing revealed that only 1–12% of the 7 CpG islands within the PTPRE promotor region are methylated and no differences in methylation status were discernible when comparing parental, chemosensitive and etoposide-resistant Y79 and WERI cells (Figure 3). Thus, PTPRE expression in RB cells is not regulated by promotor methylation.

### 2.3. Involvement of miR631 in the Regulation of PTPRE Expression

**Figure 4:**
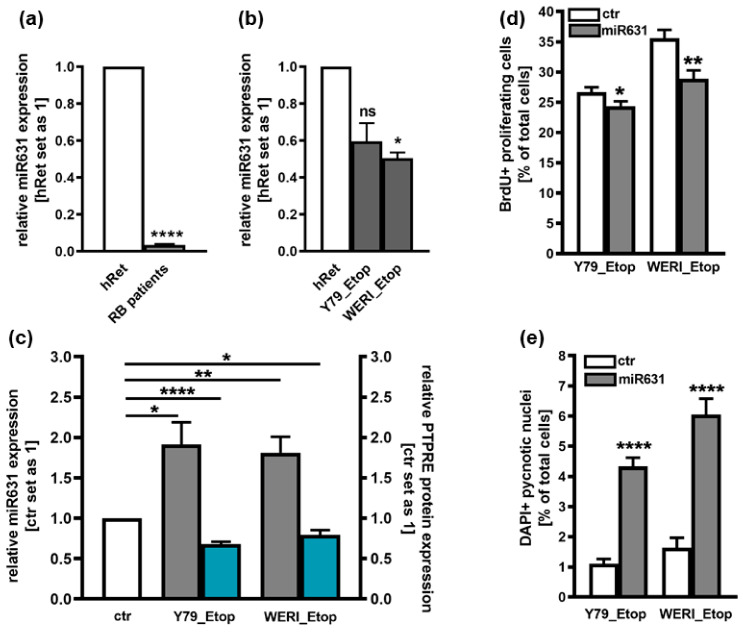
MiR631 expression in RB patient tumors (**a**) and etoposide-resistant (Etop) Y79 and WERI cell lines (**b**) as revealed by Real-time PCR and effects of miR361 overexpression (**c**) on PTPRE protein expression (**c**), proliferation (**d**) and apoptosis levels (**e**) of Y79_Etop and WERI_Etop cells. Values are means of at least three independent experiments ± SEm. ns not significant; * *p* < 0.05; ** *p* < 0.01; and **** *p* < 0.0001 statistical differences compared to the control group calculated by Student’s *t*-test.

PTPRE has been suggested as a direct downstream target of miR631 in hepatocellular carcinoma cells [17]. Attempting to decipher the mechanisms regulating the expression of PTPRE in etoposide-resistant RB cells, expression patterns of miR631 was analyzed in Y79_Etop and WERI_Etop RB cell lines and RB patient tumors. Compared to the healthy human retina, miR631 levels were significantly downregulated in RB patient tumors and in etoposide-resistant WERI_Etop cells (Figure 4a,b). Thus, miR631 and PTPRE display opposing expression patterns and we set out to test for a hypothesized regulation of PTPRE expression by this respective miR. Overexpression of miR631 resulted in significantly decreased PTPRE protein levels (Figure 4c) as well as significantly decreased proliferation rates (Figure 4d) with concomitantly upregulated apoptosis levels (Figure 4e). All effects seen mirrored those obtained following PTPRE knockdown, strongly indicating that PTPRE expression in etoposide-resistant RB is regulated by miR361.

### 2.4. Involvement of FGFb Signaling in the Regulation of PTPRE Expression

**Figure 5:**
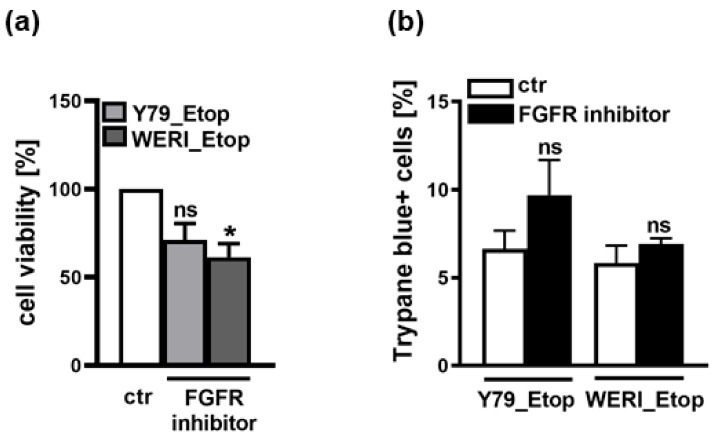
Effects of FGFR inhibition on cell viability and apoptosis of the etoposide-resistant RB cell lines Y79_Etop and WERI_Etop as revealed by WST-1 assays (**a**) and trypane blue cell counting (**b**). FGFR inhibition decreases cell viability and increases apoptosis of Y79_Etop and WERI_Etop cells compared to control cells (ctr). Values are means of three independent experiments ± SEM. ns not significant, * *p* < 0.05 statistical differences compared to the control (ctr) group calculated by Student’s *t*-test.

In a proteome profiler human oncology array, we previously found FGFb to be upregulated in etoposide-resistant RB cells. d It has already been shown that the expression of PTPRE is induced by FGF [18] and that RB cells express all four known FGF receptors (FGFR; [22]). Therefore, we hypothesized that an inhibition of the FGFR, prohibiting binding of FGFb, potentially results in decreased PTPRE expression, whereas treatment with recombinant FGF (rFGF) possibly increases PTPRE expression. Thus, we performed FGFR inhibitor studies or treated the cells with rFGF. Inhibition of the FGFR with Pemigatinib resulted in significantly decreased cell viability (Figure 5a) and slightly, yet not significantly increased apoptosis levels (Figure 5b) of etoposide-resistant Y79 and WERI cells, effects also seen upon PTPRE knockdown (see below). However, PTPRE protein expression was not clearly altered upon FGFR inhibition and administration of rFGF did not induce any changes in PTPRE levels independent of the treatment duration and the concentration used, indicating that PTPRE expression in etoposide-resistant RB cells is most likely not regulated via the FGF signaling axis.

### 2.5. PTPRE Knockdown Influences Cell Viability, Proliferation and Growth of Etoposide-Resistant Y79 and WERI Retinoblastoma Cell Lines

**Figure 6:**
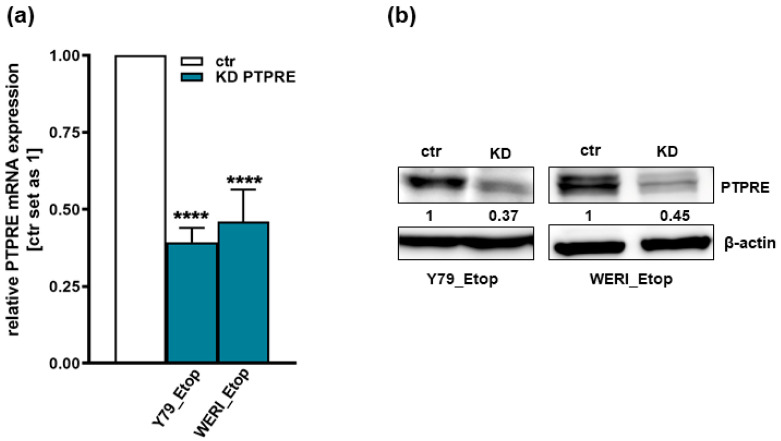
Verification of PTPRE knockdown efficiency. Efficient stable, lentiviral PTPRE knockdown (KD) in etoposide-resistant (Etop) Y79_Etop and WERI_Etop cells was verified by quantitative Real-time PCR (**a**) and Western blot analyses (**b**). Indicated numbers are intensity ratios relative to ß-actin, used as a loading control, calculated using Micro Manager 1.4 software. ctr: Y79_Etop or WERI_Etop RB cells without PTPRE knockdown, KD: Y79_Etop or WERI_Etop cells with PTPRE knockdown values are means of three independent experiments ± SEM. **** *p* < 0.0001 statistical differences compared to the control (ctr) group calculated by Student’s *t*-test.

We performed lentiviral PTPRE knockdown experiments in the etoposide-resistant RB cell lines Y79 and WERI, both exhibiting high endogenous PTPRE expression levels (Figure 1b). Efficient PTPRE knockdown levels were confirmed by quantitative Real-time PCR (Figure 6a) and Western blot analysis (Figure 6b).

**Figure 7:**
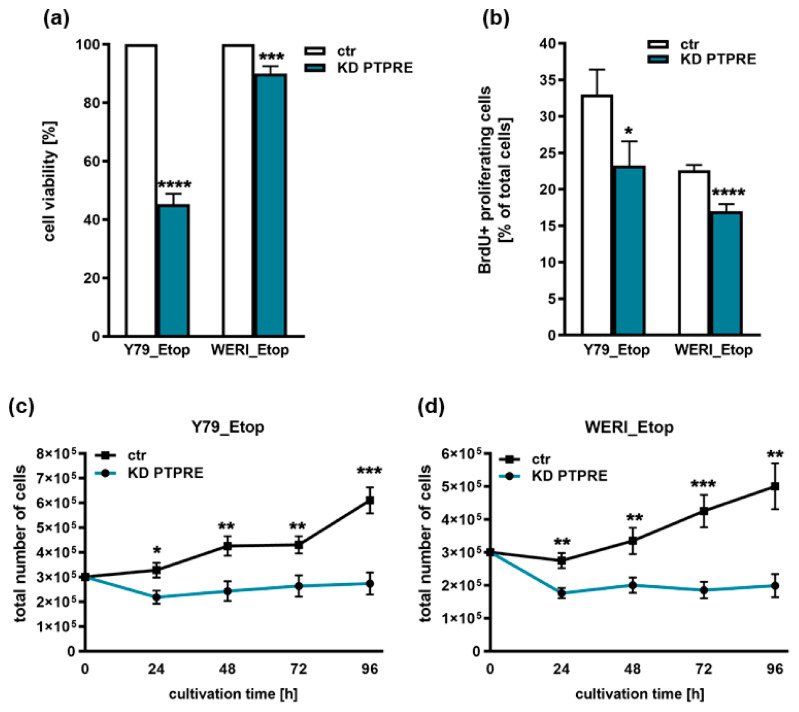
Effects of PTPRE knockdown (KD) on cell viability, proliferation and growth of the etoposide-resistant RB cell lines Y79_Etop and WERI_Etop as revealed by WST-1 assays (**a**), cell counts from BrdU stains (**b**) and growth curve analyses (**c**,**d**). Stable PTPRE knockdown decreases cell viability, proliferation levels and growth of Y79_Etop and WERI_Etop cells compared to control cells (ctr). Values are means of three independent experiments ± SEM. ns not significant; * *p* > 0.05; ** *p* < 0.01; *** *p* < 0.001 and **** *p* < 0.0001 statistical differences compared to the control (ctr) group calculated by Student’s *t*-test.

As revealed by WST-1 assays and BrdU cell counts, both etoposide-resistant RB cell lines investigated exhibited significantly lower cell viabilities (Figure 7a) and decreased proliferation rates (Figure 7b) following PTPRE knockdown. In both RB cell lines investigated, additional growth curve analyses (Figure 7c,d) after PTPRE knockdown confirmed the effects on cell proliferation shown above.

### 2.6. PTPRE Knockdown Induces Caspase Dependent Apoptosis in Etoposide-Resistant RB Cell Lines

**Figure 8:**
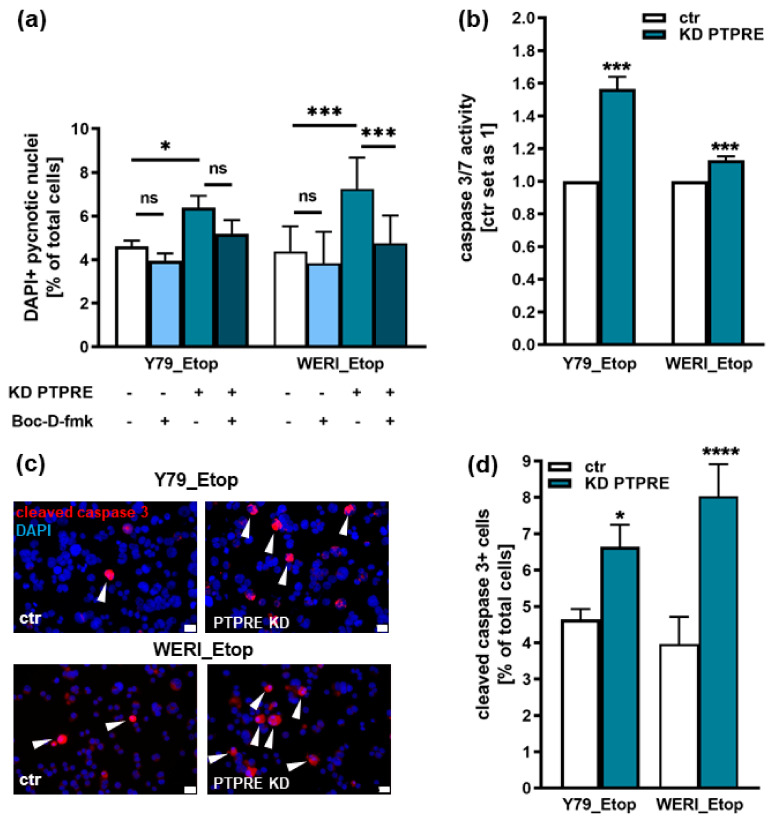
Effects of PTPRE knockdown (KD) on apoptosis levels of etoposide-resistant RB cell lines. PTPRE knockdown increases apoptosis levels of etoposide-resistant Y79 and WERI cells compared to control cells (ctr) as revealed by DAPI cell counts after treatment with the broad spectrum caspase inhibitor Boc-D-fmk (**a**), caspase3/7 assays (**b**) and immunocytochemical stains against cleaved caspase 3 (red fluorescence with blue DAPI counterstaining; arrowheads indicate cleaved caspase-3 positive (cleaved caspase-3+) cells), white scale bars: 10 µm (**c**) and quantification of cleaved caspase 3 positive cells (**d**). Values are means of three independent experiments ± SEM. ns not significant; * *p* < 0.05; *** *p* < 0.001 and **** *p* < 0.0001 statistical differences compared to the control (ctr) group calculated by Student’s *t*-test or one way ANOVA with Newman–Keuls post-test.

PTPRE knockdown resulted in a significant increase in apoptosis levels of both etoposide-resistant RB cell lines investigated (Figure 8a). To analyze whether PTPRE-induced apoptosis is caspase dependent, etoposide-resistant, PTPRE-depleted RB cells were treated with the broad-spectrum caspase inhibitor Boc-D-Fmk. Compared to cell death levels after PTPRE KD alone, apoptosis was significantly reduced after Boc-D-Fmk treatment in both cell lines, indicating the involvement of caspases in PTPRE-mediated apoptosis (Figure 8a). Additional caspase assays and immunocytochemical stains against caspase 3 revealed significantly increased caspase 3/7 activity (Figure 8b) as well as significantly increased numbers of cleaved and thereby activated caspase 3 positive cells (Figure 8c,d), confirming that PTPRE-induced apoptosis is caspase-3/7-mediated in both etoposide-resistant RB cell lines investigated.

### 2.7. PTPRE Knockdown Reduces Anchorage-Independent Growth of Etoposide-Resistant RB Cell Lines

**Figure 9:**
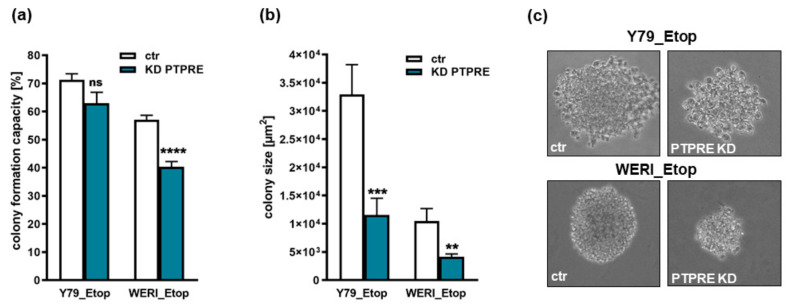
Effects of PTPRE knockdown (KD) on anchorage-independent growth of etoposide-resistant Y79 and WERI RB cells. Both etoposide-resistant (Etop) RB cell lines showed significantly reduced colony formation capacity (**a**) and colony sizes (**b**,**c**) (10× magnification) after PTPRE knockdown as revealed by soft agarose assays. Values are means of three independent experiments ± SEM. ns not significant; ** *p* < 0.01; *** *p* < 0.001 and **** *p* < 0.0001 statistical differences compared to the control (ctr) group calculated by Student’s *t*-test.

Compared to their parental counterparts, PTPRE depleted WERI_Etop cells showed a significantly reduced colony formation capacity (Figure 9a), displaying significantly smaller colonies in soft agarose assays testing for changes in anchorage-independent growth capability (Figure 9b,c). Y79_Etop cells likewise displayed significantly smaller colonies following PTPRE knockdown (Figure 9b,c), colony formation capacity did, however, not significantly change (Figure 9a).

### 2.8. PTPRE Knockdown Decreases Tumorigenicity and Migration Potential of Etoposide-Resistant RB Cells In Vivo

**Figure 10:**
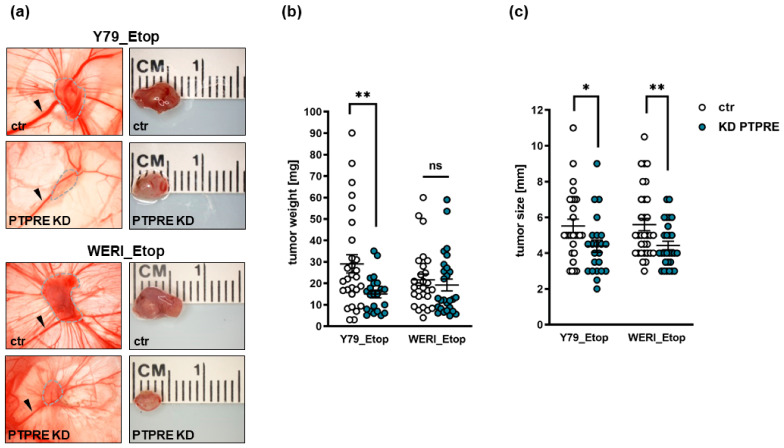
Effect of lentiviral PTPRE knockdown (KD) on tumor formation of etoposide-resistant (Etop) RB cell lines in vivo as revealed by chick chorioallantoic membrane (CAM) assays. Photographs of CAM tumors in situ and ruler measurements (in cm) of excised CAM tumors 7 days after inoculating the Y79_Etop and WERI_Etop RB cells onto the CAM. Tumor burden is marked with a dotted line and arrowheads indicate the main CAM blood vessel (**a**). The right row of pictures in (**a**) serves as scales for the tumors delineated by the dotted line in the left row of photographs. Quantification of weight (**b**) and size (**c**) of CAM tumors developing from PTPRE depleted Y79_Etop and WERI_Etop cell lines in comparison to the control cells (ctr). Values are means of at least three independent experiments ± SEM. ns not significant; * *p* < 0.05; ** *p* < 0.01 statistical differences compared to the control group calculated by Student’s *t*-test.

To investigate whether the effects of a PTPRE knockdown on etoposide-resistant RB cell growth in vitro likewise influence their tumor growth and migration potential in vivo, we used the chicken in ovo chorioallantoic membrane (CAM) assay as a model system. PTPRE-depleted Y79_Etop or WERI_Etop cells and control cells were inoculated onto the CAM of embryonic developmental day 10 (EDD10) chicken embryos. Photo-documentation of CAM tumors developing from inoculated RB cells (Figure 10a) and quantification of tumor weight (Figure 10b) and size (Figure 10c) revealed that PTPRE-depleted, etoposide-resistant RB cells develop significantly smaller tumors (Figure 10a,c) than control cells, while in the case of Y79_Etop cells, there was also significantly lower tumor weight (Figure 10b).

To exemplarily test for changes in migratory potential following PTPRE knockdown, GFP-labeled etoposide-resistant WERI-Rb1 RB cells were injected into a CAM vein. PTPRE-depleted WERI_Etop RB cells showed no reduced extravasation from the CAM vasculature into the surrounding tissue and did not displayed significant changes in the migration rate compared to their respective controls as revealed by human GAPDH real-time PCR analyses of lower CAM punches. 

### 2.9. Re-Sensitization of Resistant RB Cells towards Etoposide after PTPRE Knockdown

**Figure 11:**
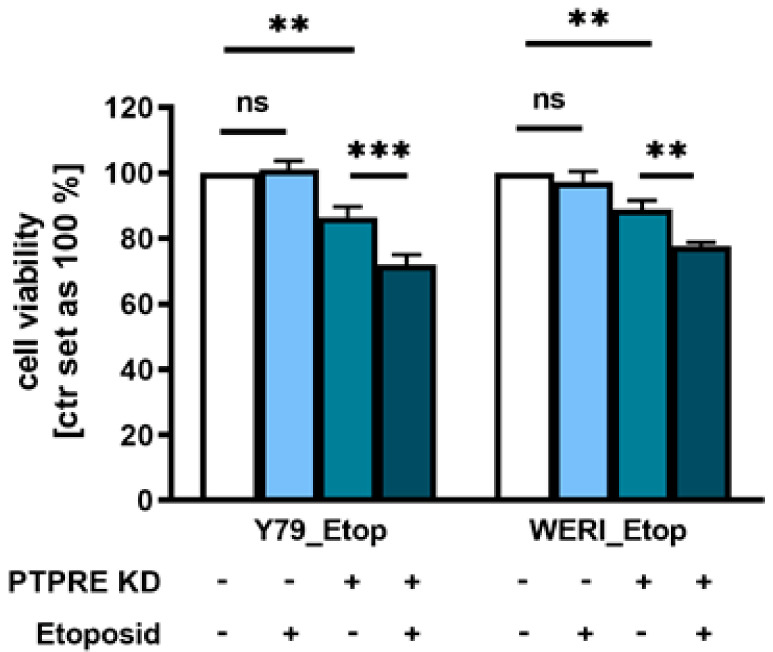
Effect of PTPRE knockdown (KD) on etoposide re-sensitization of etoposide-resistant RB cell lines. Compared to control cells, PTPRE depletion in etoposide (Etop) resistant Y79 and WERI cells induces significant changes in cell viability after etoposide treatment. Etoposide-resistant RB cell lines were treated with 3 µmol/mL (Y79_Etop) or 5 µmol/mL (WERI_Etop) etoposide for 7 days prior to WST-1 assays. Values are means of at least three independent experiments ± SEM. ns not significant; ** *p* < 0.01; *** *p* < 0.001 statistical differences compared to the control group calculated by Student’s *t*-test.

To determine whether PTPRE depletion contributes to a re-sensitization of etoposide-resistant RB cells, we again knocked down PTPRE in etoposide-resistant Y79_Etop and WERI_Etop cell lines. Treatment of PTPRE-depleted etoposide-resistant RB cell lines with etoposide did indeed result in significantly decreased cell viability (Figure 11) compared to the PTPRE knockdown alone, indicating that depletion of PTPRE increases the susceptibility of etoposide-resistant RB cells for this chemotherapeutic drug.

### 2.10. PTPRE Downstream Signaling

It has been shown that many cancer entities are connected to aberrant activation of tyrosine kinases, including PTPRE. PTPRE regulates phosphorylation processes with the involvement of different other kinases like SRC [10,20], ERK1/2 [23], glucocorticoid kinase 3 (SGK3) and AKT downstream signaling [24,25]. As all of the above-mentioned signaling molecules mediate effects related to cell proliferation, growth, viability, tumor initiation and tumor cell migration [26,27,28], effects likewise seen to be influenced following PTPRE silencing, we investigated ERK/p-ERK, SRC/pSRC, AKT/p-AKT, and SGK3/pSGK3 expression after PTPRE knockdown in order to reveal possible PTPRE downstream signaling mechanisms in etoposide-resistant RB cells.

**Figure 12:**
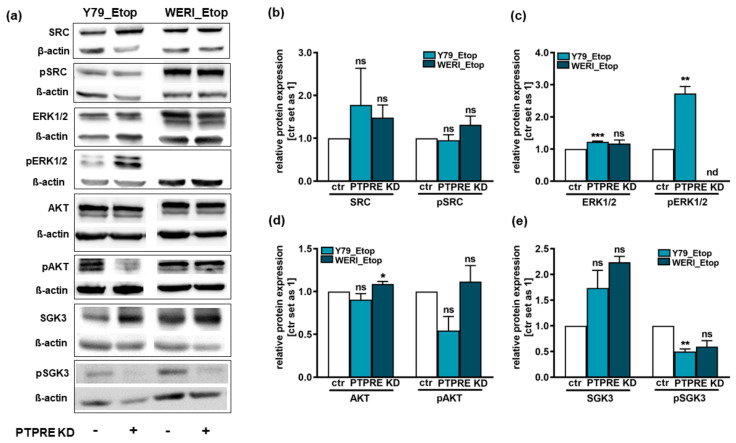
SRC, ERK1/2, AKT and SGK3 as well as the respective phospho-proteins (pSRC, pERK1/2, pAKT and pSGK3) expression levels after PTPRE knockdown (KD) in the etoposide-resistant (Etop) RB cell lines Y79_Etop and WERI_Etop as revealed by Western blot analysis. (**a**) Representative Western blots showing the expression levels of the unphosphorylated proteins SRC, ERK1/2, AKT and SGK3 and their respective phosphorylated counterparts pSRC, pERK1/2, pAKT and pSGK3 in the two etoposide-resistant RB cell lines Y79_Etop and WERI_Etop with (+) and without (−) PTPRE knockdown (KD). (**b**–**e**) Quantification of the phospho-protein/protein ratio of investigated proteins after PTPRE knockdown in etoposide-resistant Y79_Etop and WERI_Etop RB cells, normalized to ß-actin, used as a loading control. Values are means of three independent experiments ± SEM. ns not significant; * *p* < 0.05; ** *p* < 0.01 and *** *p* < 0.001; statistical differences compared to the control group calculated by paired Student’s *t*-test.

Western blot analyses revealed no changes in SRC expression or phosphorylation upon PTPRE KD in both etoposide-resistant RB lines investigated (Figure 12a,b). By contrast, in PTPRE-depleted Y79_Etop RB cells ERK1/2 as well as pERK1/2 protein levels were significantly increased (Figure 12a,c) and pAKT (Figure 12a,d) and pSGK3 levels were slightly-to-significantly decreased (Figure 12a,e). In WERI-Etop RB cells, however, changes in ERK1/2 levels were not significant and no pERK expression was detectable (Figure 12a,c). PTPRE depletion in WERI-Etop RB cells significantly increased AKT levels but did not significantly change pAKT levels (Figure 12a,d). Phospho SGK3 levels, by contrast, were likewise reduced in WERI-Etop cells (Figure 12a,e).

## 3. Discussion

Protein tyrosine phosphatases (PTPs) are enzymes that remove phosphate groups from proteins. Dysregulation of dephosphorylation can lead to uncontrolled cell growth and proliferation, apoptosis arrest and ultimately cancer development [10,12]. Initially, PTPs have been described as tumor suppressors as they terminate signaling by desphosphorylation of oncogenic kinases. However, it became clear that PTPs are overexpressed in several cancers and in this setting do not suppress tumor growth but rather promote tumor development [12]. Thus, PTPs and receptor-type protein tyrosine phosphatases (PTPRs), a subgroup of PTPs sharing a transmembrane domain [7], can act as tumor suppressors and oncogenes [7,11,12].

Our present study aimed to explore the involvement of receptor-type protein tyrosine phosphatase epsilon (PTPRE) in retinoblastoma progression in general and chemotherapy resistance in particular. PTPRE has been studied, e.g., in osteoclasts, nerve cells, and cancer cells, and it exerts different functions among various tissues (for review see: [10]). Expression of PTPRE was found to be significantly higher than average in acute myeloid leukemia (AML). PTPRE expression did, however, not display a correlation with overall AML patients’ survival [13]. No significant difference was observed in PTPRE expression in different sexes of patients, but expression displayed a weak correlation with patient age [13]. In lung adenocarcinoma, PTPRE was suggested as one of the genes potentially helpful to categorize different prognostic and therapeutic subgroups [29]. Overexpression of PTPRE was likewise reported for all stages of renal cell carcinoma [14]. Besides, PTPRE is highly expressed in thyroid cancer clinical samples and cell lines and overexpression correlates with the TNM stage [30]. Fittingly, in the study presented, we likewise observed increased PTPRE expression levels in etoposide-resistant compared to chemosensitive RB cell lines as well as in RB patient tumors compared to the healthy human retina.

To unravel molecular mechanisms potentially regulating PTPRE expression, we analyzed (i) promotor methylation of PTPRE in RB, (ii) miR631 as a putative PTPRE targeting miRand and (iii) the potential involvement of FGF receptor (FGFR) signaling.

Promoter methylation is an important mechanism for PTPR inactivation in cancer (for review see: [7]). It has been shown, that the promoter methylation status of PTPRE is strongly associated with lymph node metastasis (LNM) of papillary thyroid carcinoma (PTC) and high expression correlates with a significantly better survival [15]. Thus, PTPRE promoter methylation status has been suggested as a useful predictive biomarker of LNM in PTC [15]. Promoter hypermethylation and decreased expression level of PTPRE were described for nonfunctioning pituitary adenomas (NFPAS), whereby invasive and non-invasive NFPAs displayed only slight differences in their methylation profiles [16]. In our study presented, PTPRE promotor methylation analyses revealed no differences in methylation status when comparing chemosensitive and etoposide-resistant RB cell lines. Therefore, PTPRE expression in RB cells is not regulated by promotor methylation.

PTPRE was described as a downstream target of miR631 in hepatocellular carcinoma (HCC) [17]. MiR631 targets genes, including PTPRE, are involved in different cellular processes including proliferation, apoptosis, invasion, and drug resistance and sensitivity, therefore playing an important role in tumor progression (for review see: [31]). MiR631 levels were found to be significantly down-regulated in hepatocellular carcinoma (HCC) [17]. In prostate cancer, one of the most common cancers, the expression of miR631 is likewise low. Increasing expression levels of this miR inhibits metastasis and invasion of prostate cancer cells by inhibiting Zeta-chain-associated protein kinase 70 (ZAP70) [32]. In addition, miR631 expression is low in non-small-cell lung cancer (NSCLC) and its induction leads to an inhibition of E2F2, which regulates PI3K/AKT signaling, in turn reducing malignancy [33]. Fittingly, in the study presented, miR631 levels were likewise significantly downregulated in RB patient tumors and etoposide-resistant RB cell lines when compared to the healthy human retina. Thereby miR631 and PTPRE displayed opposing expression patterns suggesting a regulation of PTPRE expression by this miR. Overexpression of miR631 resulted in the exact same effects seen upon PTPRE knockdown, strongly indicating that PTPRE expression in etoposide-resistant RB is regulated by miR361.

RB cells were reported to express all four known FGF receptors (FGFR; [22]) and an up-regulation and activation of PTPRE was observed upon FGF stimulation of human breast cancer cells [18]. As we previously found FGFb to be upregulated in a proteome profiler oncology array of etoposide-resistant RB cells s we hypothesized that inhibition of the FGFR might result in decreased PTPRE expression, whereas treatment with recombinant FGF (rFGF) possibly increases PTPRE expression. FGFR inhibition with Pemigatinib resulted indeed in significantly decreased viability of etoposide-resistant RB cells, the effects of which were also seen upon PTPRE knockdown. FGFR inhibition did, however, not significantly decrease PTPRE expression levels. Treatment of WERI_Etop and Y79_Etop cells with recombinant FGF likewise did not induce any changes in PTPRE levels, indicating that PTPRE expression in etoposide-resistant RB cells is unlikely to be regulated via the FGF-FGFR signaling axis.

As PTPs in general and PTPRE in particular have been controversially discussed as tumor suppressors or oncogenes [7,11,12,19], in the study presented we set out to investigate the functional effects of a PTPRE knockdown in etoposide-resistant RB cell lines. Upregulated PTPRE has been shown to promote cell proliferation, whereas PTPRE KD resulted in the opposite effect indicating an oncogenic function in thyroid carcinoma [30]. Moreover, cells isolated from mammary tumors induced by *Neu* in mice genetically lacking PTPRE proliferated less than corresponding mammary tumor cells isolated from mice expressing the phosphatase [9]. Further along this line, reduced PTPRE levels decreased the viability and anchorage-independent growth of human breast cancer cells [18]. These findings are in good accordance with data from our study presented, showing significantly reduced cell viability, growth and proliferation upon PTPRE KD in etoposide-resistant RB cell lines, indicating an oncogenic role of PTPRE in retinoblastoma. In addition, the PTPRE depletion also led to a re-sensitization of the resistant RB cell lines towards etoposide, underlining its potential role in cancer cell transformation processes [10].

It has been shown that PTPRE expression in murine mammary glands leads to massive hyperplasia and associated tumorigenesis [9] and high PTPRE expression in thyroid cancer correlates with tumor size [30]. In accordance with these findings, in our study presented, PTPRE-depleted, etoposide-resistant RB cells developed significantly smaller tumors in ovo than control cells, supporting the notion of PTPRE being an oncogene rather than a tumor suppressor in RB. PTPRE-depleted etoposide-resistant WERI-Rb1 cells did, however, not display significant changes in migration rates in ovo, contradicting findings that PTPRE promotes migration in macrophages [34] and acts as a metastatic promoter in hepatocellular carcinoma [35].

In order to investigate putative downstream signaling pathways, we set out to analyze the protein expression and phosphorylation status of the four known downstream targets of PTPRE SRC, ERK1/2, AKT and SGK3. It has been reported that at the molecular level, PTPRE dephosphorylates and thereby activates SRC [9,19,20]. Activation of SRC has been shown to activate downstream pathways like, e.g., the PI3K-AKT pathway, playing an essential role in cell proliferation, survival, growth, tumor initiation, metastasis, and drug resistance (for review see: [26]). The activity of SRC is significantly reduced in tumor cells lacking PTPRE and restoring SRC activity revealed that it is only its reduced activity that caused aberrant proliferation rates of tumor cells lacking PTPRE [9]. Further along this line, the lack of PTPRE increased SRC phosphorylation at its C-terminal inhibitory Tyr^527^ sites by 51% in Neu-induced *Ptpre^−/−^* murine mammary carcinoma tumor cells [19]. By contrast, our study revealed that although PTPRE KD slightly increased pSRC levels in etoposide-resistant WERI-Rb1 cells, the effects did not reach significance, indicating that the anti-tumorigenic effects of a PTPRE KD in etoposide-resistant RB cells are not mediated via this downstream mediator.

Previous studies suggested an involvement of ERK1/2 in PTPRE downstream signaling [18,23,30]. The serotonin-tyrosine kinase ERK, phosphorylated and thereby activated by MEK, primarily plays a role in promoting cell proliferation and anti-apoptotic effects (for review see: [27]). Unexpectedly, silencing of PTPRE expression in human breast cancer cells has been shown to abolish ERK1/2 activation, leading to significantly diminished pERK levels [18] and siPTPRE-transfected thyroid cancer cells likewise exhibited decreased pERK1/2 levels [30]. In line with these findings, ectopic expression of PTPRE in MCF-7 cell lines resulted in activation of ERK1/2 [18] and pERK levels were clearly increased in thyroid cancer cells upon PTPRE upregulation [30]. By contrast, phosphorylation and thereby activation of ERK1/2 was reduced after PTPRE transfection of non-carcinogenic HEK293T cells [23]. Fittingly, in our study significantly increased pERK levels were observed upon PTPRE KD in Y79_Etop RB cells. Upregulated pERK1/2 levels do, however, not fit the anti-proliferative and anti-survival effects seen after PTPRE KD in these cells. Besides, in WERI-Etop RB cells, no pERK expression was detectable. Thus, the ERK/pERK signaling is most likely not involved in mediating PRPRE effects in RB cells.

Retinoblastoma cells have been described to activate the AKT pathway [36]. Phosphorylated and thereby activated AKT is known to be implicated in regulation of cell survival, growth and apoptosis (for review see: [28]). As PTPRE was found to contribute to AKT activation [18], we hypothesize that PTPRE might signal via the AKT axis in RB cells as well. In the study presented, PTPRE KD did not change pAKT levels in etoposide-resistant WERI-Rb1 cells but resulted in slightly yet not significantly decreased pAKT levels in etoposide-resistant Y79 RB cells. Fittingly, PTPRE silencing in MCF-7 and MDA-MB-231 cells abolished AKT activation by significantly diminishing pAKT levels [18] and siPTPRE-transfected thyroid cancer cells likewise exhibited decreased pAKT levels [30]. However, as PTPRE is a phosphatase, dephosphorylation of its targets should be abolished or at least diminished upon its silencing, resulting in increased rather than decreased pAKT levels. Thus, although reduced pAKT levels would fit reduced cell proliferation and growth seen in PTPRE-depleted etoposide-resistant RB cells, these effects cannot be directly mediated via PTPRE.

The PI3K-dependent serum and glucocorticoid inducible protein kinase 3 (SGK3) shares a substrate specificity to AKT and has been suggested to play a critical role in AKT-independent oncogenic signaling (for review see: [37]). It has been shown that in tumor cells with minimal AKT activation, SGK3 conferred increased cell viability [37]. Fittingly, in the study presented, the effects of PTPRE depletion on pAKT levels did not reach significance, whereas pSGK3 levels were significantly decreased in etoposide-resistant Y79 RB cells and likewise reduced in WERI_Etop cells. However, as already discussed above, PTPRE is a phosphatase, and thus, one would expect an increase rather than a decrease in pSGK3 levels upon its depletion. Nevertheless, PTPRE downstream effects might be mediated indirectly via the AKT-independent SGK3 axis rather than the AKT pathway.

Summarizing, one can state that PTPRE acts as an oncogene in RB and is most likely involved in chemotherapy resistance mechanisms by enhancing cell proliferation and tumor growth and inhibiting apoptosis. Although molecular signaling pathways remain to be elucidated in more detail in the future, miR631 seems to be a key player in the regulation of PTPRE in RB. As small molecules and antibodies inhibiting the activity of tyrosine kinases are effective tools in cancer treatment [38], regulators of tyrosine kinase activity like the tyrosine phosphatase PTPRE hold the potential of new future RB therapy targets.

## 4. Materials and Methods

### 4.1. Human Retina and Retinoblastoma Samples

Postmortem healthy human retina (hRet) and patient RB samples were used for comparative expression studies. The study methodologies conformed to the standards set by the Declaration of Helsinki. The Ethics Committee of the Medical Faculty of the University of Duisburg-Essen approved the use of human retina (approval # 06-30214) and RB samples (approval # 14-5836-BO) for research conducted in the course of the study presented and written informed consent has been obtained from patients’ relatives or parents.

### 4.2. Cell Lines and Culture

The human retinoblastoma (RB) cell lines RB355, Rbl-13, Rbl30, RB247, R383 [39], Y79 [40] and WERI-Rb1 (WERI) [41] as well as the corresponding etoposide-resistant RB cell lines Y79_Etop and WERI_Etop were kindly provided by Dr H. Stephan. RB cell lines were cultivated as described previously [42]. Human embryonic kidney cells (HEK293T) were grown as adherent cell culture in DMEM (PAN-Biotech, Aidenbach, Germany) with 10% FBS (PAN-Biotech, Aidenbach, Germany), 4 mM L-glutamine (Gibco, Karlsruhe, Germany), 100 U penicillin/mL, and 100 µg streptomycin/mL (Gibco, Karlsruhe, Germany) at 37 °C, 5% CO_2_ and 95% humidity. No approval from an ethics committee was required for work with the human cell lines.

### 4.3. Lentiviral PTPRE Knockdown

In order to generate lentiviral particles, 6 × 10^6^ human embryonic kidney cells (HEK293T) were transfected with 6 µg of each of the following plasmid DNAs: (I) packaging vectors pczVSV-G [43] and pCD NL-BH [43], (II) shPTPRE clone#198 (clone TRCN0000315198) for transduction of Y79_Etop cells, (III) shPTPRE clone #895 (clone TRCN0000002895) for transduction of WERI_Etop Rb1 cells, (V) pPRIME-CMV-Neo-FF3 (p234) as a negative control for all knockdown experiments or (VI) GFP expression vector (pCL7EGwo) each in the presence of 45 µg polyethyleneimine (PEI, branched, Sigma-Aldrich, St. Louis, MO, USA) in DMEM medium. After 24 h, the medium was changed to Iscove’s Modified Dulbecco´s medium (IMDM, Pan-Biotech, Aidenbach, Germany) with 10% FBS and 1% penicillin/streptomycin and 72 h after transfection viral supernatants were harvested, filtered (0.45 µm filter) and cryoconserved.

For stable transduction, 1.25 × 10^6^ RB cells were seeded in DMEM medium. After 24 h, the medium was removed and the cells were transducted with PTPRE virus particles or control virus particles each in the presence of polybrene (5 µL per mL lentivirus; H9268, Sigma-Aldrich, München, Germany). After 24 h, DMEM medium with supplements (twice the volume of the virus particles) was added and 48 h later the medium was completely changed, and the cells were incubated for another 72 h.

### 4.4. PTPRE Promotor Analysis

Genomic DNA from all RB cell lines were isolated using the DNeasy Blood and Tissue Kit (Qiagen, Hilden, Germany) and bisulfite-converted with the EZ DNA Methylation-Gold Kit (Zymo Research, Freiburg, Germany) to convert all cytosines to uracil, while the methylated cytosines remain unmodified. PTPRE promoter sequences were amplified from bisulfite-converted DNA by PCR using the following protocol: 5 min at 95 °C, 50 cycles of 1 min 95 °C, 2 min 53/54 °C, 1 min 72 °C followed by final 10 min 72 °C step. PCR forward and reverse primer sequences were as follows: PTPRE (for) 5′-GTGAGTGTTTGTTATATTTTGATTTTT-3′ and PTPRE (rev) 5′-CTATCCTCAACCTAACAAAAAATTTA-3′. Afterwards, the PCR product was purified from agarose gels by Illustra GFX PCR DNA and Gel Band Purification Kit (VWR Lifescience, Darmstadt, Germany) and subcloned into the pCR^®^4-TOPO^®^ vector using a TOPO TA Cloning Kit for Sequencing (Invitrogen, Karlsruhe, Germany). Plasmid isolation was performed with the GeneJET Plasmid Miniprep Kit (Thermo Scientific, Oberhausen, Germany) and individual clones were sequenced by Microsynth using the M13 forward and reverse primer. Sequences of at least 5 clones were analyzed using the Clone Manager 9 software to identify methylated cytosine residues.

### 4.5. Plasmids and miR631 Overexpression

To generate a miR631 expression vector (pSG5-miR631), the human miR631 sequence was amplified from cDNA of HEK293T cells by RT-PCR using the forward primer 5′-CGGAATTCAACAGGCAGAGATCAGAGGG-3′ and the reverse primer 5′-CGGGATCCTGTCGGGATTACAGGTGTGG-3′ containing EcoRI and BamHI restriction sites. The PCR product was cloned into the pCRII-TOPO vector (Invitrogen, Karlsruhe, Germany), excised by digestion with EcoRI and BamHI and ligated into the pSG5 vector (#216201, Stratagene, La Jolla, CA, USA) digested with the same restriction enzymes. The empty pSG5 vector was used as control and transfection was performed as described previously [44]. A total of 24 h after transfection, the cells were harvested and used for protein and RNA isolation.

### 4.6. FGF Receptor Inhibitor Studies

For FGF receptor inhibition studies, 7.5 × 10^6^ cells were seeded in 25 mL medium and incubated with 100 µM FGFR inhibitor (Pemigatinib INCB054828; MedChemExpress, Monmouth Junction, NJ, USA) for 120 h. DMSO (Sigma-Aldrich, München, Germany)-treated cells served as a solvent control.

### 4.7. Re-Sensitization Studies

To study the re-sensitizing effects of PTPRE depletion on the chemosensitivity of etoposide-resistant RB cells, 1 × 10^6^ Y79_Etop and WERI_Etop cells were seeded in 2 mL DMEM with supplements with or without etoposide (3 µmol/mL or 5 µmol/mL for Y79_Etop and WERI_Etop, respectively) in a 6-well plate and cultivated at 37 °C. A total of 1 mL DMEM with supplements was added after 96 h of cultivation. After 7 days, 4 × 10^4^ cells were seeded in a 96-well plate in 100 µL DMEM with supplements with or without etoposide (3 µmol/mL or 5 µmol/mL for Y79_Etop and WERI_Etop, respectively) in triplicates. A total of 10 µL of a water-soluble tetrazolium (WST-1) salt solution (Sigma-Aldrich, München, Germany) was added to each well and cells were incubated for a designated period at 37 °C. Formazan dye produced by viable cells was quantified by measuring the absorbance in a microplate reader at 450 nm (Agilent BioTek, Santa Clara, CA, USA).

### 4.8. RNA Extraction and Quantitative Real-Time PCR

RNA isolations from RB cells and CAM tissue were performed using the NucleoSpin^®^ RNA II Kit (Macherey & Nagel, Düren, Germany) and the miRNeasy Kit (Qiagen, Hilden, Germany), respectively. For quantitative Real-time PCR analyses, cDNA was synthesized with the QuantiTect Reverse Transcription Kit (Qiagen, Hilden, Germany) according to the manufacturer’s protocol. For analysis of PTPRE expression, a SYBR^TM^ green PCR assay (Applied Biosystem, Darmstadt, Germany) was used with specific primers for PTPRE 5′-CTGAGCCAACTGGATGGAAT-3′ (forward) and 5′-TTGGGACCTTGAGCTGCTAT-3′ (reverse) as well as 5′-ACCCACTCCTCCACCTTTGA-3′ (forward) and 5′-CTGTTGCTGTAGCCAAATTCGT-3′ (reverse) for human *GAPDH* (h*GAPDH*) as an endogenous control. Real-time PCR reactions were conducted in triplicates in 20 µL of SYBR^TM^ green PCR Mastermix (Applied Biosystem, Darmstadt, Germany) performing 40 cycles of the following program: 95 °C for 15 min, 94 °C for 15 s, 55 °C for 30 s and 70 °C for 34 s. For micro-RNA expression analyses, a miScript PCR Starter Kit (# 2181193; Qiagen, Hilden, Germany) was used, following the manufacturer´s protocol. The designated miScript HiSpec Buffer (Qiagen, Hilden, Germany) for quantification of mature miRNA was used along with specific primers for miR631 5′-AGACCTGGCCCAGACCTCAGC-3′ and *5.8S* RNA (5′-CTACGCCTGTCT GAGCGTCGCTT-3′) as an endogenous control. The reactions were performed in duplicates using a QuantStudio^TM^ 3 real-time PCR system (Thermo Fisher, Darmstadt, Germany), with the following program: 95 °C for 15 min, 94 °C for 15 s, 55 °C for 30 s and 70 °C for 34 s and 40 cycles. RNA isolation from chicken chorioallantoic membrane (CAM) tissue punches (see below) was performed as described previously [45]. Quantitative real-time PCR analyses were performed and quantified following the protocol published previously [45].

### 4.9. Western Blotting

For Western blot analyses, cells were washed in phosphate-buffered saline (PBS, PAN-Biotech GmbH, Aidenbach, Germany) and lysed in radioimmunoprecipitation assay (RIPA) buffer plus supplements [46] for 30 min at 4 °C on a shaker and centrifuged at 10,000 rpm at 4 °C for 30 min. Afterwards, protein concentrations were measured by bicinchoninic acid assay (BCA; Thermo-Scientific, Oberhausen, Germany) according to the manufacturer’s protocol. Equal amounts of protein extracts were separated on a 10% SDS-PAGE and transferred onto nitrocellulose membranes. Membranes were incubated with primary antibodies at 4 °C overnight (Table 1).

HRP-conjugated species-specific secondary antibodies (goat-anti-rabbit; P0448 and rabbit-anti-mouse; P0260; DAKO, Glostrup, Denmark) were used in dilutions of 1:10,000 at room temperature for 1 h on a tabletop shaker. The signals were developed by adding Western Bright Chemiluminescence Reagent (Cytiva, Buckinghamshire, UK).

### 4.10. Cell Viability Assays

For cell viability assays, 4 × 10^4^ cells in 100 µL medium were seeded in a 96-well plate in two triplicates. After different incubation times depending on the experimental settings, 10 µL of a water-soluble tetrazolium (WST-1) salt solution (Sigma-Aldrich, München, Germany) was added to each well and cells were incubated at 37 °C for a designated period. Quantification of the formazan dye produced by viable cells was performed by measuring the absorbance at 450 nm in a microplate reader (Agilent BioTek, Santa Clara, CA, USA).

### 4.11. Growth Kinetic

To determine growth kinetics in a 24-well plate format, 3 × 10^5^ cells were seeded in 500 µL DMEM medium (PAN-Biotech, Aidenbach, Germany) with supplements in triplicates and the number of vital cells was counted manually every 24 h (5 time points: 0 h, 24 h, 48 h, 72 h and 96 h) in a Neubauer chamber using the trypan blue exclusion method.

### 4.12. Cell Proliferation and Apoptosis Detection

Cell proliferation was determined by 5-Bromo-2′-deoxyuridine (BrdU; Sigma, Hamburg, Germany) incorporation. For BrdU immunochemistry, 4 h prior to PFA fixation 5 µM BrdU was added to the cells. Afterwards, cells were permeabilized and incubated with a rat anti-BrdU antibody (1:1000; ab6326; Abcam, Cambridge, UK) and the BrdU signal was visualized using a goat anti-rat secondary antibody labelled with Alexa Fluor 594 (1:1000; Molecular Probes, Eugene, OR, USA). For each experiment, five coverslips were stained, and the percentages of proliferating cells were calculated by setting BrdU-positive cells in relation to the total amount of DAPI-positive cells.

### 4.13. Caspase Dependent Apoptosis

In order to inhibit endogenous caspase activity, the broad-spectrum caspase inhibitor Boc-D-Fmk (Calbiochem, Dramstadt, Germany) was used. Cells were seeded on poly-D-lysin coated coverslips as described before and were cultured in the presence of 50 µL Boc-D-Fmk for 24 h. Afterwards, the cells were fixed with 4% PFA and DAPI stained to determine the number of pycnotic nuclei. To analyze caspase 3 and 7 cleavage activity after PTPRE knockdown, a caspase-Glo 3/7 assay (Promega, Madison, WI USA) was used. Therefore, 1.5 × 10^5^ PTPRE-depleted and control cells were seeded in 500 µL growth medium supplemented with 2% FBS in a 24-well plate format and incubated overnight. After 24 h, 60 µL of each cell suspension was mixed with 60 µL of caspase-3/7 reagent and seeded in a white 96-well plate for 2 h at room temperature protected from light. Afterwards, luminescence was measured with an Orion II microplate luminometer (Berthold Detection Systems, Pforzheim, Germany) following the manufacturer´s instructions. Measurements were performed three times in five replicates. Cleaved, activated caspase 3 was detected immunocytochemically using a rabbit monoclonal cleaved caspase 3 antibody (#9664; 5A1E; Cell Signalling, Danvers, MA, USA) diluted 1:400 in PBS with 0.1% Triton™ X-100, 4% BSA and 1% NGS at 4 °C overnight in a humidified chamber after 1 h PFA fixation and methanol permeabilisation. A goat anti-rabbit antibody, labelled with Alexa-fluor^®^594 (Molecular Probes, Eugene, OR, USA), diluted 1:1000 in PBS with 1% BSA, was used to visualize cleaved caspase 3 positive cells via fluorescent microscopy with a Nikon Eclipse E600 microscope (Nikon, Düsseldorf, Germany).

### 4.14. Colony Formation and Soft Agarose Assay

Soft agarose assays were performed as described previously [43]. A total of 5000 cells were seeded in 2 mL soft agarose in a six-well dish in triplicates and cultivated for 3 weeks. Colony formation capacity (%) was calculated by counting the number of colony-forming cells and viable single cells in six visual fields (10× magnification) in triplicates per assay. Colony size was measured by capturing images using a Nikon Eclipse TS2 microscope equipped with a digital camera and IC MEASURE 1.0 software (Nikon, Düsseldorf, Germany). For the determination of colony size, eight colonies per well were measured.

### 4.15. CAM Assays

In order to study the effects of PTPRE knockdown on tumor formation and migration capacity in vivo, PTPRE depleted etoposide-resistant RB and control cells were inoculated on the extraembryonic chorioallantoic membrane (CAM) of chicken embryos on EDD10 mainly following the protocols published by Zijlstra and Palmer [47,48]. Ten eggs were inoculated with 1 × 10^6^ cells suspended in 50 µL PBS in at least three independent experiments. Seven days after grafting, at EDD17, grown tumors were excised, measured, weighted and photographed as described previously [45].

Intravenous injection of GFP-labeled Y79_Etop and WERI_Etop control and PTPRE knockdown cells was carried out on EDD12 as described previously by our group [45]. Five days after injection (EDD17), the chicken embryos were sacrificed and six punches of the ventral CAM, opposing the injection site, were collected and processed as described previously [48,49]. Successful migration after injection was monitored by the identification of the GFP-labelled cells via fluorescence microscopy of the CAM punches. RNA isolations and quantification of hGAPDH of the pooled CAM punches tissue were performed as described previously [45].

Wholemount immunofluorescent staining of CAM vessels was carried out as described previously [50]. The first antibody-detecting chicken desmin (D33; ab8470, Abcam, Cambridge, UK) was diluted 1:20 in PBS with 3% BSA and incubated in a humidified chamber at 4 °C overnight. Alexa-fluor^®^594 goat anti mouse IgG (Molecular Probes, Eugene, OR, USA) was diluted 1:1000 in 300 µL of PBS and CAM punches were incubated in a 24-well plate format on a shaker overnight at 4 °C. Subsequent fluorescence microscopy was carried out with a Nikon ECLIPSE E600 microscope and NIS Elements Imaging 5.20.02 software (Nikon, Düsseldorf, Germany).

### 4.16. Immunohistochemistry

For PTPRE immunohistochemical localization in formalin fixed, paraffin embedded retinoblastoma and to immunohistochemically localize GFP labeled cells in fixed and embedded CAM tumors, sample sections were deparaffinized and steamed for 1 h in EDTA buffer (pH 9 for PTPRE and GFP) to improve antigen retrieval. Sections were analyzed as previously described [51] following the manufacturers description of the Vectastain Elite ABC kit (Biozol, Eching, Germany). Immunostaining was performed using a rabbit monoclonal antibody against PTPRE (#PA5-82543; Invitrogen, Thermofisher Scientific) and GFP (2955S; Cell Signaling Technology, Danvers, MA, USA) at a dilution of 1:200 (PTPRE and GFP) in antibody dilution buffer provided in the Vectastain kit (Biozol, Eching, Germany) at 4 °C overnight in a humidified chamber. As controls, in all cases, antibody dilution buffer was substituted for the primary antisera to test for nonspecific labeling. No specific cellular staining was observed when the primary antiserum was omitted. Images were acquired by a slide scanner (Leica, Wetzlar, Germany) and viewed using the Aperio Image Scope Software 12.3 (Leica).

### 4.17. Statistical Analysis

All assays were performed at least in triplicates. Statistical analyses were performed using GraphPad Prism 9. Data represent means ± SEM of three independent experiments from independent RB cell cultures. Results were analyzed by a Student’s *t*-test and considered significantly different if *p*-value < 0.05 (*), *p*-value < 0.01 (**), *p*-value < 0.001 (***) or *p*-value < 0.0001 (****). Statistics on the growth curves was performed using a free web interface http://bioinf.wehi.edu.au/software/compareCurves/ accessed on 1 March 2021, which uses the “compare growth curves” function from a statistical modeling package called statmod, available from the “R Project for Statistical Computing”: http://www.r-project.org accessed on 1 March 2021, previously described elsewhere [52].

## Figures and Tables

**Table 1:** Primary antibodies for Western blot analyses.

Antibodies	Company	Dilution
PTPRE (Ma5-25072)	Thermofisher Scientific, Darmstadt, Germany	1:1000
SRC (#2105)	Cell signaling technology, Danvers, MA, USA	1:1000
pSRC (#2123)	Cell signaling technology, Danvers, MA, USA	1:1000
ERK (#9102)	Cell signaling technology, Danvers, MA, USA	1:1000
pERK (#4370)	Cell signaling technology, Danvers, MA, USA	1:1000
AKT (#4685)	Cell signaling technology, Danvers, MA, USA	1:1000
pAKT (#9271)	Cell signaling technology, Danvers, MA, USA	1:500
SGK3 (Sc-166847)	Santa Cruz Biotechnology, Dallas, TX, USA	1:500
pSGK3 (#5642)	Cell signaling technology, Danvers, MA, USA	1:500
FGFb (ab215373)	Abcam, Berlin, Germany	1:1000
ß-actin (#4967)	Cell signaling technology, Danvers, MA, USA	1:1000

## Data Availability

The original contributions presented in the study are included in the article, further inquiries can be directed to the corresponding author.
